# Impact of plasma potassium normalization on short-term mortality in patients with hypertension and hypokalemia or low normal potassium

**DOI:** 10.1186/s12872-020-01654-3

**Published:** 2020-08-24

**Authors:** Maria Lukács Krogager, Peter Søgaard, Christian Torp-Pedersen, Henrik Bøggild, Christina Ji-Young Lee, Anders Bonde, Jesper Q. Thomassen, Gunnar Gislason, Manan Pareek, Kristian Kragholm

**Affiliations:** 1 Department of Cardiology, Aalborg University Hospital, Aalborg, Denmark; 2 Department of Cardiology and Clinical Research, Nordsjællands Hospital, Hillerød, Denmark; 3 Public Health and Epidemiology Group, Department of Health Science and Technology, Aalborg University, Aalborg, Denmark; 4 Unit of Epidemiology and Biostatistics, Aalborg University Hospital, Aalborg, Denmark; 5 Department of Cardiology, Copenhagen University Hospital, Herlev and Gentofte, Hellerup, Denmark; 6 Department of Clinical Biochemistry, Rigshospitalet, University of Copenhagen, Copenhagen, Denmark; 7 Department of Cardiology, Herlev and Gentofte University Hospital, Hellerup, Denmark; 8 The Danish Heart Foundation, Copenhagen, Denmark; 9 The National Institute of Public Health, University of Southern Denmark, Copenhagen, Denmark; 10 Department of Internal Medicine, Yale New Haven Hospital, Yale University School of Medicine, New Haven, USA; 11 Brigham and Women’s Hospital, Heart & Vascular Center, Harvard Medical School, Boston, USA; 12 Department of Cardiology, Regionshospital Nordjylland, Hjørring, Denmark

**Keywords:** Hypokalemia, Borderline hypokalemia, Hypokalemia correction, Mortality, Low potassium.

## Abstract

**Background:**

Hypokalemia is common in patients treated with antihypertensive drugs, but the impact of correcting hypokalemia is insufficiently studied. We examined the consequences of hypokalemia and borderline hypokalemia correction in patients with hypertension.

**Methods:**

We identified 8976 patients with hypertension and plasma potassium concentrations ≤3.7 mmol/L within 100 days from combination antihypertensive therapy initiation. The first measurement between 6 and 100 days after the episode with potassium ≤3.7 mmol/L was retained. We investigated all-cause and cardiovascular mortality within 60-days from the second potassium measurement using Cox regression. Mortality was examined for seven predefined potassium intervals derived from the second measurement: 1.5–2.9 mmol/L (*n* = 271), 3.0–3.4 mmol/L (*n* = 1341), 3.5–3.7 (*n* = 1982) mmol/L, 3.8–4.0 mmol/L (*n* = 2398, reference), 4.1–4.6 mmol/L (*n* = 2498), 4.7–5.0 mmol/L (*n* = 352) and 5.1–7.1 mmol/L (*n* = 134).

**Results:**

Multivariable analysis showed that potassium concentrations 1.5–2.9 mmol/L, 3.0–3.4 mmol/L, 4.7–5.0 mmol/L and 5.1–7.1 mmol/L were associated with increased all-cause mortality (HR 2.39, 95% CI 1.66–3.43; HR 1.36, 95% CI 1.04–1.78; HR 2.36, 95% CI 1.68–3.30 and HR 2.62, 95% CI 1.73–3.98, respectively). Potassium levels <3.0 and > 4.6 mmol/L were associated with increased cardiovascular mortality. The adjusted standardized 60-day mortality risks in the seven strata were: 11.7% (95% CI 8.3–15.0%), 7.1% (95% CI 5.8–8.5%), 6.4% (95% CI 5.3–7.5%), 5.4% (4.5–6.3%), 6.3% (5.4–7.2%), 11.6% (95% CI 8.7–14.6%) and 12.6% (95% CI 8.2–16.9%), respectively.

**Conclusions:**

Persistent hypokalemia was frequent and associated with increased all-cause and cardiovascular mortality. Increase in potassium to levels > 4.6 mmol/L in patients with initial hypokalemia or low normal potassium was associated with increased all-cause and cardiovascular mortality.

## Novelty and Significance

What is new?

- Correcting plasma potassium concentrations ≤3.7 mmol/L to levels between 3.5–4.6 mmol/L was associated with improved short-term prognosis

What is relevant?

- Increased mortality risk was observed in patients who initially had borderline hypokalemia, partly because they developed hypokalemia. This emphasizes that potassium supplementation might be relevant in patients with low normal potassium concentrations
- Correcting hypokalemia and borderline hypokalemia shortly was associated with good prognosis
- Low potassium concentrations have previously been associated with arrhythmogenesis and increased mortality risk in patients with hypertension.

Summary

In this register based study we investigated the impact of correcting hypokalemia and borderline hypokalemia on 60-day mortality among 8976 patients treated with combination antihypertensive therapy. We observed that: (1) persistent hypokalemia was common and associated with increased all-cause and cardiovascular mortality; (2) Increase in potassium to levels > 4.6 mmol/L in patients with initial hypokalemia or low normal potassium was associated with increased all-cause and cardiovascular mortality; (3) Among patients with borderline hypokalemia initially, development of hypokalemia or hyperkalemia was associated with increased mortality risk; (4) Correcting hypokalemia associated with increased survival.

## Background

Several common clinical conditions and drugs are known to cause or precipitate hypokalemia [1]. Among patients with hypertension, thiazides are the antihypertensive drugs most frequently associated with hypokalemia [2–4].

We recently demonstrated a U-shaped relationship between potassium levels and mortality among patients with hypertension. We observed an increased mortality risk even in patients with low and high normal serum potassium concentrations, suggesting a narrower than previously thought normal interval for potassium of 4.1–4.7 mmol/L. [5] However, at present there is no evidence regarding the consequences of potassium normalization in patients with hypertension and hypokalemia. Therefore, it is essential to examine how correction and even overcorrection of hypokalemia affect prognosis in patients with hypertension.

Using Danish national registers, we investigated the 60-day mortality among patients with hypertension and hypokalemia or low normal potassium concentrations, according to their subsequent plasma potassium concentrations measured within 6–100 days following the initial episode with low potassium levels.

## Methods

### Data sources

In Denmark, a unique and personal identification number is allocated to all individuals at the time of birth or immigration. This unique identifier allows linkage of health and administrative data at the individual level [6] and ensures nearly complete follow-up. We used anonymized data from five different registers made available by Statistics Denmark after central encryption of the unique identifiers [7]. An overview of the registers used in this study is available in Supplementary Table S1. In Denmark, register-based studies using anonymized data provided by Statistics Denmark are not warranted approval from the ethics committee.

### Study population

We defined hypertension as redemption of minimum two antihypertensive agents in two consecutive quarters. This definition has previously been validated [8]. Patients entered the present study in the second quarter, referred to as the date of hypertension. An overview of the Anatomical Therapeutic Chemical Classification System (ATC) codes used to identify patients with hypertension is available in Supplementary Table S2. We required a plasma potassium measurement ≤3.7 mmol/L within 100 days from the date of hypertension for inclusion. The first measurement within this time interval was retained and referred to as the first potassium measurement (K_1_). The second potassium measurement (K_2_) was identified in the interval 6–100 days from K_1_ and the first draw within this timeframe was retained. We did not include potassium concentrations within 1–5 days from K_1_ as, potassium disarrays are usually corrected within a few days, regardless of the strategies applied. Patients below 18 years of age were excluded from the study. Supplementary Figure S1 illustrates the population flowchart.

### Comorbidities and medication

We identified comorbidities and medications regarded as confounders when studying the association between changes in potassium levels and short-term mortality. The following comorbidities dated up to 5 years before the index date (K_2_ date) were identified: hospitalization for heart failure, ischemic heart disease, stroke, chronic obstructive pulmonary disease, chronic liver disease, diabetes mellitus, inflammatory bowel disease and malignancy. Furthermore, patients with a past history of primary adrenal insufficiency, primary hyperaldosteronism, and diabetes insipidus were excluded. The International Classification of Disease (ICD) codes used to identify above-mentioned comorbidities are shown in Supplementary Table S3. We used the Chronic Kidney Disease Epidemiology Collaboration (CKD-EPI) equation [9] to calculate renal function, and an estimated glomerular filtration rate (eGFR) < 30 mL/min/1.73 m^2^ described significant renal insufficiency. Patients were excluded if no creatinine concentrations were available the same day as or within a week from the index date. Patients with missing serum sodium measurements on the index date were also excluded.

Prescriptions redeemed up to 90-days before the index date were identified for the following drugs: potassium supplements, non-steroidal anti-inflammatory drugs, corticosteroids, laxatives, xanthines, and antimicrobials. See Supplementary Table S3 for relevant ATC codes.

### Exposure variable

Serum and plasma measurements yield similar results, but for serum samples there is a risk of contamination with potassium from burst platelets during coagulation in the range of 0.1–0.5 mmol/L due to non-standard sample handling [10]. Therefore, we only used plasma potassium measurements.

There is not a consensus on the normal plasma potassium interval, as it can vary from population to population. Supplementary Table S4 gives an overview on the three most used reference intervals in serum and plasma originating from different populations. We defined hypokalemia as plasma potassium concentrations below 3.5 mmol/L and borderline hypokalemia as potassium levels within the interval 3.5–3.7 mmol/L. Hyperkalemia was defined as potassium levels above 4.6 mmol/L. [11] For K_2_, seven predefined potassium intervals were constructed: 1.5–2.9 mmol/L, 3.0–3.4 mmol/L, 3.5–3.7 mmol/L, 3.8–4.0 mmol/L, 4.1–4.6 mmol/L, 4.7–5.0 mmol/L and 5.1–7.1 mmol/L. Plasma potassium interval K: 3.8–4.0 mmol/L was used as the reference for statistical analyses. We chose this interval as the reference group because it had one of the largest number of patients and lowest mortality rate.

### Outcome

The primary outcome was all-cause mortality within 60 days from K_2_. The secondary outcome was presumed cardiovascular death within 60 days from K_2_.

### Statistical analyses

Categorical variables were presented as counts and percentages, and continuous variables as median with corresponding 25th and 75th percentiles. Differences between variables were compared using chi-squared and Kruskal-Wallis tests, as appropriate.

To illustrate survival probability, Kaplan Meier curves were plotted for the seven potassium intervals. A restricted cubic spline curve was constructed to investigate the relationship between potassium as a continuous variable and absolute mortality risk in an age, sex, comorbidity and drug standardized population.

Cox proportional hazard modeling was used to analyze the association between the seven predefined potassium intervals and 60-day all-cause and presumed cardiovascular mortality. Based on the Cox regression principle, we modelled an average effect to estimate the 60-day absolute risk of all-cause mortality, with potassium interval 3.8–4.0 mmol/L as reference.

The multivariable model was adjusted for: age, sex, serum sodium, renal insufficiency, malignancy, heart failure, chronic liver disease, chronic obstructive pulmonary disease, diabetes mellitus, stroke, atrial flutter/fibrillation, ischemic heart disease, inflammatory bowel disease, antihypertensive therapy, corticosteroids, antimicrobials, non-steroidal anti-inflammatory drugs, xanthines, laxatives, and potassium supplements. The proportional hazard assumption was tested by plotting Schoenfeld residuals and was not violated. Interactions on mortality were tested by comparing the likelihood ratio of the Cox regression model with and without the interaction term. The following variables were tested for interaction with plasma potassium on mortality: age, sex, and renal insufficiency. A two-sided *p*-value < 0.01 was considered statistically significant for interactions. We found no statistically significant interactions. For other analyses, a two-sided *p*-value < 0.05 was considered statistically significant. Linearity of age on mortality was also assessed through a likelihood ratio test comparing a linear description with a categorical one. Age was found to violate linearity and was included as a categorical variable with five levels, using cut-off values from every 20th percentiles (55, 64, 72, 79 and 101 years). Hazard ratios (HR) and absolute risks (AR) were estimated with 95% confidence intervals (95% CI). All data management and analyses were performed using SAS, version 9.4 and R, version 3.5.0 [12].

## Results

### Demographics

We identified 8976 patients treated with combination antihypertensive therapy who had potassium concentrations ≤3.7 mmol/L within the first 100 days from combination therapy initiation. Baseline characteristics for the cohort stratified on the seven predefined K_2_ intervals are presented in Table 1. Females accounted for 53% of the total population and median age was 68.3 years (range 18.2–100.8 years). Of the patients with borderline hypokalemia at K_1_, 13% developed hypokalemia and 5.7% hyperkalemia at K_2_. As for patients with hypokalemia at K_1_, we observed that 28.5% remained hypokalemic at the second blood draw and 4.8% developed hyperkalemia. Approximately half of the population was hospitalized at K_1_ and four fifths at K_2_. See supplementary Figure S2 displaying the distribution of K_1_, average of potassium measurements drawn within 1–5 days from K_1_, and K_2_. A low number of patients (*n* = 572) had renal insufficiency at the time of second potassium draw. Median time from K_1_ to K_2_ was 22 days (range: 6–100 days). As for diuretic treatment, thiazides were common in patients with potassium concentrations ≤3.7 mmol/L, whereas loop diuretics were more common among patients with high potassium levels. Thiazide-like diuretics accounted for 4.4% of the total prescriptions of thiazides.

**Table 1:** Demographics stratified according to the eight predefined plasma potassium intervals in a cohort of 8976 patients treated with combination antihypertensive therapy

		1.5–2.9 mmol/L (***n*** = 271)	3.0–3.4 mmol/L (***n*** = 1341)	3.5–3.7 mmol/L (***n*** = 1982)	3.8–4.0 mmol/L (***n*** = 2398)	4.1–4.6 mmol/L (***n*** = 2498)	4.7–5.0 mmol/L (***n*** = 352)	5.1–7.1 mmol/L (***n*** = 134)	***p***-value
Age	median (range)	70.6(21.4, 94.9)	67(19.2, 100.8)	67(22.1, 97.7)	67.7(19.2, 97.5)	69.7(18.2, 99.9)	69.9(20.2, 98.6)	71.9(27.7, 97.8)	< 0.01
Sex	Female	157 (57.9)	757 (56.5)	1087 (54.8)	1276 (53.2)	1250 (50.0)	172 (48.9)	57 (42.5)	
Renal insufficiency (second measuremt)		26 (9.6)	78 (5.8)	118 (6.0)	127 (5.3)	142 (5.7)	43 (12.2)	38 (28.4)	< 0.01
Serum sodium (second measurement)	median (range)	138(111, 157)	139(117, 179)	140(105, 155)	140(101, 161)	139(107, 159)	138(114, 166)	136(112, 149)	< 0.01
Plasma potassium (first measurement)	3.5–3.7 mmol/L	97 (35.8)	700 (52.2)	1322 (66.7)	1768 (73.7)	1877 (75.1)	258 (73.3)	89 (66.4)	
	< 3.5 mmol/L	174 (64.2)	641 (47.8)	660 (33.3)	630 (26.3)	621 (24.9)	94 (26.7)	45 (33.6)	< 0.01
Renal insufficiency (first measuremt)		25 (9.8)	87 (6.9)	117 (6.2)	127 (5.5)	166 (6.9)	47 (13.7)	32 (24.8)	< 0.01
	missing creatinine	17	77	92	82	77	8	5	
Hospitalization at the time of second potassium measurement		232 (85.6)	1050 (78.4)	1538 (77.7)	1860 (77.6)	2050 (82.1)	303 (86.1)	118 (88.1)	< 0.01
Time from first to second potassium measurement (days)	median (range)	14(6, 97)	21(6, 100)	25(6, 100)	26(6, 100)	21(6, 100)	13.5(6, 100)	14(6, 97)	< 0.01
Death-60 days		39 (14.4)	94 (7.0)	125 (6.3)	124 (5.2)	168 (6.7)	48 (13.6)	29 (21.6)	< 0.01
60-day cardiovascular mortality		21 (7.7)	50 (3.7)	68 (3.4)	59 (2.5)	91 (3.6)	28 (8.0)	14 (10.4)	< 0.01
**Comorbidities**									
Any malignancy		58 (21.4)	252 (18.8)	389 (19.6)	429 (17.9)	478 (19.1)	71 (20.2)	28 (20.9)	0.66
Chronic obstructive pulmonary disease		41 (15.1)	180 (13.4)	238 (12.0)	290 (12.1)	395 (15.8)	63 (17.9)	28 (20.9)	< 0.01
Diabetes		43 (15.9)	221 (16.5)	324 (16.3)	432 (18.0)	453 (18.1)	77 (21.9)	36 (26.9)	0.01
Chronic kidney disease		16 (5.9)	116 (8.7)	176 (8.9)	170 (7.1)	194 (7.8)	38 (10.8)	26 (19.4)	< 0.01
Chronic liver disease		23 (8.5)	55 (4.1)	90 (4.5)	109 (4.5)	124 (5.0)	21 (6.0)	12 (9.0)	0.01
Atrial fibrillation/Atrial flutter		37 (13.7)	209 (15.6)	337 (17.0)	446 (18.6)	535 (21.4)	85 (24.1)	43 (32.1)	< 0.01
Hypertension (ICD-10)		112 (41.3)	475 (35.4)	755 (38.1)	895 (37.3)	941 (37.7)	121 (34.4)	35 (26.1)	0.04
Heart failure		47 (17.3)	212 (15.8)	348 (17.6)	453 (18.9)	624 (25.0)	102 (29.0)	59 (44.0)	< 0.01
Ischemic heart disease		54 (19.9)	257 (19.2)	420 (21.2)	594 (24.8)	729 (29.2)	126 (35.8)	53 (39.6)	< 0.01
Stroke		36 (13.3)	147 (11.0)	241 (12.2)	292 (12.2)	320 (12.8)	40 (11.4)	13 (9.7)	0.67
Inflammatory bowel disease		4 (1.5)	19 (1.4)	43 (2.2)	39 (1.6)	38 (1.5)	5 (1.4)	6 (4.5)	0.12
**Pharmacotherapy**									
Potassium supplement	ATC: A12B	163 (60.1)	699 (52.1)	895 (45.2)	1083 (45.2)	1219 (48.8)	192 (54.5)	80 (59.7)	< 0.01
	ATC: C03	67 (24.7)	415 (30.9)	557 (28.1)	674 (28.1)	577 (23.1)	67 (19.0)	13 (9.7)	< 0.01
Antimicrobials		152 (56.1)	742 (55.3)	1082 (54.6)	1347 (56.2)	1434 (57.4)	204 (58.0)	78 (58.2)	0.59
Beta-2 agonists		58 (21.4)	337 (25.1)	451 (22.8)	576 (24.0)	625 (25.0)	93 (26.4)	38 (28.4)	0.30
Corticoids		54 (19.9)	284 (21.2)	417 (21.0)	499 (20.8)	534 (21.4)	81 (23.0)	33 (24.6)	0.90
Laxatives		7 (2.6)	41 (3.1)	51 (2.6)	58 (2.4)	74 (3.0)	23 (6.5)	4 (3.0)	< 0.01
Xantines		11 (4.1)	43 (3.2)	57 (2.9)	56 (2.3)	81 (3.2)	15 (4.3)	5 (3.7)	0.26
NSAIDs		153 (56.5)	739 (55.1)	1090 (55.0)	1362 (56.8)	1438 (57.6)	197 (56.0)	76 (56.7)	0.67
Antiadrenergic drugs		4 (1.5)	21 (1.6)	18 (0.9)	26 (1.1)	26 (1.0)	≤3	4 (3.0)	0.24
Vasodilators		0 (0.0)	0 (0.0)	≤3	0 (0.0)	0 (0.0)	0 (0.0)	0 (0.0)	0.74
Beta blockers		97 (35.8)	497 (37.1)	775 (39.1)	972 (40.5)	1213 (48.6)	174 (49.4)	71 (53.0)	< 0.01
Calcium channel blockers		117 (43.2)	514 (38.3)	687 (34.7)	843 (35.2)	780 (31.2)	93 (26.4)	38 (28.4)	< 0.01
Renin angiotensin system inhibitors		134 (49.4)	716 (53.4)	1158 (58.4)	1487 (62.0)	1506 (60.3)	209 (59.4)	81 (60.4)	< 0.01
Loop diuretics		115 (42.4)	533 (39.7)	720 (36.3)	841 (35.1)	1112 (44.5)	179 (50.9)	87 (64.9)	< 0.01
Thiazide diuretics		127 (46.9)	682 (50.9)	934 (47.1)	1110 (46.3)	909 (36.4)	95 (27.0)	20 (14.9)	< 0.01
Thiazilde-like diuretics		7 (2.6)	28 (2.1)	46 (2.3)	47 (2.0)	37 (1.5)	4 (1.1)	≤3	0.38
Potassium sparing diuretics		15 (5.5)	40 (3.0)	50 (2.5)	54 (2.3)	48 (1.9)	7 (2.0)	≤3	< 0.01
Mineral receptor antagonists		51 (18.8)	181 (13.5)	250 (12.6)	281 (11.7)	372 (14.9)	83 (23.6)	35 (26.1)	< 0.01

*ICD-10* International Classification of Disease 10th version, *ATC* Anatomical Therapeutic Chemical Classification System, *NSAIDs* Non-steroidal anti-inflammatory drugs

≤3- Is ascertained cells where the frequency is 1, 2 or 3 in order to ensure anonymization of the data

Demographics stratified on survival status showed that age, renal insufficiency, lower sodium concentrations, hospitalization at the time K_1_, prior history of malignancy, chronic liver disease, chronic obstructive pulmonary disease, atrial fibrillation/atrial flutter, heart failure, and stroke were predominant among the deceased (Supplementary Table S5).

### 60-day survival after the second potassium measurement

**Fig. 1:**
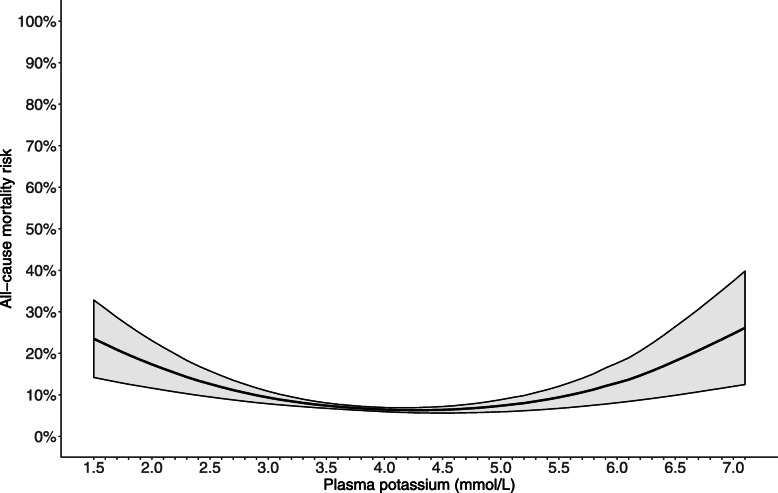
Age, sex, comorbidity and drug standardized 60-day risk of all-cause death in relation to plasma potassium as a continuous variable. Model adjusted for age, gender, plasma sodium, renal insufficiency, malignancy, heart failure, chronic liver disease, chronic obstructive pulmonary disease, diabetes mellitus, atrial flutter/fibrillation, stroke and ischemic heart disease, antihypertensive therapy, corticosteroids, antimicrobials, non-steroidal anti-inflammatory drugs, potassium supplement, xanthines, laxatives

During 60-day follow-up after K_2_, 627 (7.0%) patients died, 331 from a cardiovascular cause. Mortality in the seven strata was: 14.4, 7.0, 6.3, 5.2, 6.7, 13.6 and 21.6%, respectively. The restricted cubic spline curve revealed a U-shaped relationship between potassium and mortality (Fig. 1).

**Fig. 2:**
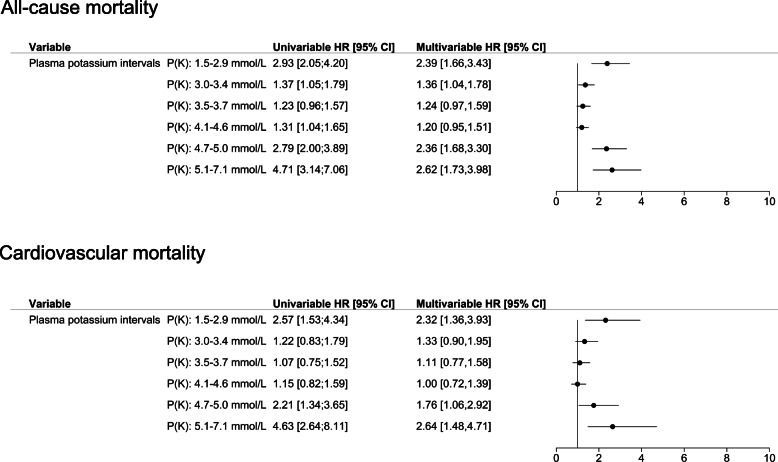
All-cause and cardiovascular mortality after hypokalemia or borderline hypokalemia according to subsequent potassium measurements in patients treated with combination antihypertensive therapy (60-days follow-up, *n* = 8976). Potassium interval K: 3.8–4.0 mmol/L represented the reference range. Adjusted for age, gender, serum sodium, renal insufficiency, malignancy, heart failure, chronic liver disease, chronic obstructive pulmonary disease, diabetes mellitus, atrial flutter/fibrillation, stroke and ischemic heart disease, antihypertensive therapy, corticosteroids, antimicrobials, non-steroidal anti-inflammatory drugs, potassium supplement, xanthines, laxatives

The results of the multivariable Cox regression, with plasma potassium 3.8–4.0 mmol/L as the reference group are shown in Fig. 2. All-cause mortality was significantly increased in patients with hypokalemia (1.5–2.9 mmol/L HR 2.39, 95% CI 1.66–3.43 and 3.0–3.4 mmol/L HR 1.36, 95% CI 1.04–1.78) when compared with the reference. We observed a trend towards increased mortality in patients with borderline hypokalemia and with potassium levels within the interval 4.1–4.6 mmol/L (HR 1.24, 95% CI 0.97–1.59 and HR 1.20, 95% CI 0.95–1.51, respectively). All-cause mortality was also elevated in patients with hyperkalemia (4.7–5.0 mmol/L HR 2.36, 95% CI 1.68–3.30; 5.1–5.7 mmol/L HR 2.62, 95% CI 1.73–3.98). The univariable analysis showed similar results. We observed no interaction between K_1_ and K_2_ on 60-day mortality.

Cardiovascular mortality accounted for nearly 53% of all deaths. We observed increased risk of cardiovascular death in patients with initial hypokalemia or low normal potassium levels who had potassium concentrations < 3.0 mmol/L and > 4.6 mmol/L at the second measurement.

The standardized 60-day absolute risk of all-cause mortality was lowest in patients with potassium concentrations between 3.8–4.0 mmol/L (AR 5.4, 95% CI 4.5–6.3%, Table 2). Significant differences in risks (reported against the reference) were observed for the following potassium intervals: 1.5–2.9 mmol/L risk difference 6.3% (95% CI 2.9–9.7%); 4.7–5.0 mmol/L risk difference 6.2% (95% CI 3.2–9.3%); 5.1–7.1 mmol/L risk difference 7.2% (95% CI 2.8–11.6%).

**Table 2:** 60-day standardized absolute risk for all-cause death after hypokalemia or borderline hypokalemia according to subsequent potassium measurements in patients treated with combination antihypertensive therapy (*n* = 8976). Potassium interval K: 3.8–4.0 mmol/L represented the reference range. Adjusted for age, gender, serum sodium, renal insufficiency, malignancy, heart failure, chronic liver disease, chronic obstructive pulmonary disease, diabetes mellitus, atrial flutter/fibrillation, stroke and ischemic heart disease, antihypertensive therapy, corticosteroids, antimicrobials, non-steroidal anti-inflammatory drugs, potassium supplement, xanthines, laxatives

	Absolute risk %, (95% CI)	60-d Risk difference %, (95%CI)	*p*-value	Average risk ratio %, (95%CI)	*p*-value
P(K) 1.5–2.9 mmol/L	11.7% (8.3–15.0)	6.3 (2.9–9.7)	< 0.001	2.17 (1.46–2.88)	0.001
P(K) 3.0–3.4 mmol/L	7.1% (5.8–8.5)	1.7 (0.1–3.4)	0.03	1.32 (0.99–1.66)	0.06
P(K) 3.5–3.7 mmol/L	6.4% (5.3–7.5)	1.0 (− 0.3–2.4)	0.14	1.19 (0.91–1.47)	0.17
P(K) 3.8–4.0 mmol/L	5.4% (4.5–6.3)	REF.		REF.	
P(K) 4.1–4.6 mmol/L	6.3% (5.4–7.2)	0.9 (−0.3–2.2)	0.13	1.18 (0.92–1.44)	0.17
P(K) 4.7–5.0 mmol/L	11.6% (8.7–14.6)	6.2 (3.2–9.3)	< 0.001	2.17 (1.51–2.82)	< 0.001
P(K) 5.1–7.1 mmol/L	12.6% (8.2–16.9)	7.2 (2.8–11.6)	0.001	2.34 (1.45–3.22)	0.003

### Subgroup and sensitivity analyses

We performed eleven additional analyses to test the accuracy and robustness of the main results (Table S6).

First, multivariable analysis performed on a subgroup of patients without kidney insufficiency showed that potassium levels within the intervals 1.5–2.9 mmol/L and 3.0–3.4 mmol/L were associated with increased mortality risk compared with the reference (3.8–4.0 mmol/L) (HR 2.33, 95% CI 1.56–3.46 and HR 1.35, 95% CI 1.02–1.79, respectively).

Second, in a subpopulation without history of malignancy, adjusted analyses showed that potassium concentrations outside the interval 3.0–4.6 mmol/L were associated with increased risk of death compared with the reference.

Third, subgroup analysis on patients without history of heart failure and no loop diuretic prescription showed that patients with hypokalemia and hyperkalemia had an increased mortality risk compared with patients with potassium levels in the interval 3.8–4.0 mmol/L.

Fourth, analysis performed on a subgroup of patients without ischemic heart disease showed that patients with severe hypokalemia, and hyperkalemia had increased risk short-term mortality risk when compared with the reference.

Fifth, looking at patients with borderline hypokalemia at the first potassium measurement, we observed that patients who developed hypokalemia (1.5–2.9 mmol/L: HR 2.16, 95% CI 1.25–3.73; 3.0–3.4 mmol/L: HR 1.70, 95% CI 1.22–2.37), or hyperkalemia (4.7–5.0 mmol/L: HR 1.84, 95% CI 1.18–2.86; 5.1–7.1 mmol/L: HR 2.81, 95% CI 1.68–4.71) had an increased risk of death within 60-days when compared with the reference.

Sixth, among patients with hypokalemia at K_1_, analyses showed that potassium concentrations within the intervals 1.5–2.9 mmol/L, 4.1–4.6 mmol/L and 4.7–5.0 mmol/L were associated with increased short-term mortality risk.

Seventh, by performing the analyses on the last available potassium measurement within 6–100 days from K_1_ instead of the first measurement, we noted that potassium levels below 3.8 mmol/L were associated with increased short-term mortality.

Eighth, analyses on patients with available K_2_ measurements within 6–45 days from K_1,_ showed that severe hypokalemia and hyperkalemia were associated with 60-day all-cause mortality.

Ninth, analyses on patients with available K_2_ measurements above 45 days from K_1,_ showed that potassium interval 3.0–3.4 mmol/L was associated with 60-day all-cause mortality.

Tenth, we stratified K_2_ in three intervals: 1.5–3.4 mmol/L (hypokalemia), 3.5–4.6 mmol/L (normokalemia) and 4.7–7.1 mmol/L (hyperkalemia). Mortality within 60-days was increased both in patients with hypokalemia (HR 1.36, 95% CI 1.12–1.66) and in patients with hyperkalemia (HR 2.13, 95% CI 1.66–2.74) at K_2_ measurement compared with patients with normal potassium concentrations.

Eleventh, multivariable analysis on patients with available magnesium measurements at the time of plasma potassium draws, showed significant association of potassium levels below 3.0 mmol/L and mortality (HR 2.46, 95% CI 1.05–5.74). In addition, we also observed a trend towards increased risk of death in patients with potassium between 3.0–3.4 mmol/L.

## Discussion

This Danish register-based cohort study investigated 60-day mortality among 8976 patients with hypertension and hypokalemia or low normal potassium in relation to a subsequent potassium measurement. The major findings were: (1) Persistent hypokalemia following low potassium was more than twice as frequent as development of hyperkalemia. (2) Persistent hypokalemia was common and associated with increased all-cause and presumed cardiovascular mortality; (3) Increase in potassium to levels > 4.6 mmol/L in patients with initial hypokalemia or low normal potassium was associated with increased all-cause and cardiovascular mortality; (4) Among patients with borderline hypokalemia initially, development of hypokalemia or hyperkalemia was associated with increased mortality risk; (5) Correcting hypokalemia associated with increased survival.

In the current study, we observed significantly higher 60-day mortality risk in patients with potassium concentrations < 3.5 or > 4.6 mmol/L after an episode with hypokalemia or low normal potassium. This finding was not surprising as we previously observed an apparent optimal potassium range within 4.1–4.7 mmol/L in a similar population [5]. Of 8976 patients with initial plasma potassium ≤3.7 mmol/L, 18% had potassium concentrations ≤3.7 mmol/L at the second measurement and 5.4% > 4.6 mmol/L, suggesting that potassium deficit is frequently underestimated than overestimated by physicians. Notably, 13% of the patients with borderline hypokalemia (K: 3.5 and 3.7 mmol/L) at the first measurement experienced a further decrease in potassium (< 3.5 mmol/L) at the second measurement. This suggests that the association of low normal potassium concentrations with mortality that we previously observed [5] can partly be explained by further declines in potassium levels, and that low normal potassium concentrations might be a marker for an ongoing decrease in potassium.

Our results also suggest that correction of hypokalemia is important in relation to short-term mortality, as patients in the middle of the normal reference interval had good prognosis. Guidelines recommend supplementation with potassium when plasma potassium levels are below 3.5 mmol/L. [13] However, in this study we cannot elucidate because of the low follow-up time the mechanism through which patients increased or decreased in potassium concentrations. It is also difficult to state whether potassium is a risk factor or a risk marker regarding mortality. Our population is relatively old, patients are treated with at least two antihypertensive drugs, and about 20% of the patients have history of heart failure, ischemic heart disease, atrial fibrillation/flutter, chronic obstructive pulmonary disease and diabetes. Possibly, potassium concentrations at non-cardiotoxic levels more likely are a risk marker of great disease burden, which is very important to recognize and identify.

Potassium supplementation of asymptomatic patients with low normal concentrations is controversial. Guidelines in the US recommend a stricter standard for potassium replacement therapy (< 4.0 mmol/L) especially in patients with cardiovascular disease who are at high risk of ventricular tachyarrhythmias [13]. Our study suggests that potassium concentrations in the middle of the reference interval are beneficial even in patients with potassium levels ≤3.7 mmol/L.

Various studies have previously demonstrated that hypokalemia among patients with cardiovascular disease is associated with an increased mortality risk [14–18]. However, no prior studies have investigated the impact of potassium normalization on short-term survival. Though, one study examined the impact of correcting hypokalemia within 24 h on the risk of cardiac arrhythmias in hospitalized patients without coronary syndromes or history of arrhythmias [19]. The authors did not find increased odds of arrhythmia in patients with hypokalemia whose potassium levels were not corrected ≥3.5 mmol/L. Although, the study does not describe or account for the cause of admission, comorbidities or pharmacotherapy. The investigators excluded patients with history of ischemic heart disease and arrhythmia, but included patients with heart failure who have a high arrhythmia risk. Overall, both the study population and the outcome measure differed in this paper compared with our study.

Another study performed on 5916 individuals from the general population found no significant associations between borderline hypokalemia (3.4–3.6 mmol/L) and risk of all-cause mortality, risk of stroke or risk of acute myocardial infarction [20]. Comparing the results of our study with this study is difficult due to major differences in study population, methodology and aim. First, our population was characterized by redemption of at least two antihypertensive drugs. Mattsson et al. [20] enrolled participants from the general population, where 49.6% had high blood pressure at baseline, 13.9% were prescribed heart medication and 10.9% were treated with diuretics. In our population, we observed higher burden of cardiovascular disease and use of diuretics. Second, our aim was to investigate the impact of correcting hypokalemia or borderline hypokalemia on short-term all-cause and cardiovascular mortality. In terms of mortality, Mattsson et al. [20] followed participants from their fourth examination in 2001–2003 until November 2014 or death, having a median follow-up of 11.9 years (Q1-Q3: 11.4–12.5 years). As potassium is varying over time especially in patients with cardiovascular disease or treated with antihypertensive drugs, use of one potassium measurement to assess mortality over more than 10 years can provide results that are difficult to interpret. Shorter follow-up time or time varying analysis where the authors accounted for both multiple measurements over time and change in relevant medication would have provided better methodology. Although, it is important to acknowledge that correcting hypokalemia and low normal potassium might not have the same impact in general population compared to a population with heart disease.

Another study investigated the influence of dyskalemia at admission and early dyskalemia correcting on short-term survival and cardiac events among intensive care unit (ICU) patients [21]. The authors concluded that patients with persisting hypokalemia or hyperkalemia within the first 2 days in ICU had increased risk of death. The two populations are not comparable, however both studies emphasize the importance of rapid correction of hypokalemia to improve short-term mortality.

### Limitations

The limitations are related to the observational nature of register-based studies, which imply non-causal interpretation of the results.

We did not have information about comorbidities and risk factors from the primary sector. Therefore, patients who did not redeem any medication of interest or were not registered an ICD-code from the secondary sector could have been misclassified as “healthy”. Patients with complications related to hypertension have a larger likelihood for being referred to the secondary sector and therefore also a higher probability for being diagnosed with other conditions (compared with patients with uncomplicated hypertension), leading to an ascertainment/surveillance bias and non-differential misclassification bias. To reduce this bias, we defined hypertension as use of at least two antihypertensive drugs in two concomitant quarters. Whether hypertension was resistant, controlled or uncontrolled was unkown, and data about ejection fraction and type of heart failure was not available.

We cannot exclude that the blood draws may contain hemolysis. However, in case of significant hemolysis the samples submitted are rejected and no potassium value is available.

We could not investigate the effect of any potential treatment or drug dosage adjustment in the time between the first and second potassium measurement. The Danish National Prescription Registry records filled prescriptions; thus, changes in dosage cannot be identified, unless a new drug is prescribed. In addition, the majority of the patients were hospitalized at the time of potassium measurement and any treatment during hospitalization is not registered in the Danish National Prescription Registry. Moreover, it was also difficult to identify the cause of hypokalemia using the registers. Hypokalemia might have occurred due to administration of diuretics, alkalosis, derangements in the renin angiotensin aldosterone system, gastroenteritis or other pathologies. However, the purpose of this study was neither to investigate the cause of hypokalemia, nor to assess the strategies used to correct low potassium concentrations. The purpose of this study was to find a clue, whether normalization of potassium had an effect on short-term mortality, whether we should increase potassium concentrations in patients with borderline hypokalemia and whether potassium actually increased.

It is also important to acknowledge that plasma potassium is not always a good predictor of the whole body potassium. Yet, it is the most commonly used method to assess potassium and only in patients with persistent hypokalemia over a longer period of time total body potassium is calculated.

## Conclusion

Persistent hypokalemia was frequent and associated with increased all-cause and cardiovascular mortality. Increase in potassium to levels > 4.6 mmol/L in patients with initial hypokalemia or low normal potassium was associated with increased all-cause and cardiovascular mortality.

### Perspectives

We were not able to report the initiatives medical doctors undertook after observing potassium levels below 3.8 mmol/L at the first measurement. However, our results emphasize the importance of potassium normalization after an episode with hypokalemia and low normal potassium and that overcorrection is associated with an increased risk of death. Potassium concentrations in the middle of the normal reference interval are associated with good prognosis. Possibly, potassium supplementation, use of mineral receptor antagonists or thiazide-like diuretics instead of thiazide-type in patients with potassium concentrations ≤3.7 mmol/L could be of clinical importance, but requires further study, preferably through a randomized controlled trial.

## Supplementary information

**Additional file 1.**

## Data Availability

Due to restrictions related to Danish law and protecting patient privacy, the combined set of data used in this study can only be made available through a trusted third party, Statistics Denmark. This state organisation holds the data used for this study. University-based Danish scientific organisations can be authorized to work with data within Statistics Denmark and such organisation can provide access to individual scientists inside and outside of Denmark. Data are available upon request to authorized scientists by contacting Statistics Denmark: http://www.dst.dk/en/OmDS/organisation/TelefonbogOrg.aspx?kontor=13&tlfbogsort=sektion or the Danish Data Protection Agency: https://www.datatilsynet.dk/english/the-danish-data-protection-agency/contact/. More information regarding data access is available at https://www.dst.dk/en/TilSalg/Forskningsservice.

## Abbreviations

ATC: Anatomical therapeutic chemical classification system
K_1_: First potassium measurement within 100 days from combination antihypertensive therapy initiation
K_2_: First potassium measurement within 6–100 days from K_1_
ICD: International classification of disease
CKD-EPI: Chronic kidney disease epidemiology collaboration
eGFR: Estimated glomerular filtration rate
HR: Hazard ratio
AR: Absolute risk
95% CI: 95% confidence interval
NSAIDs: Non-steroidal anti-inflammatory drugs
