# Supplementary material for: Impact of plasma potassium normalization on short-term mortality in patients with hypertension and hypokalemia or low normal potassium
Source: BMC Cardiovasc Disord. 2020 Aug 24;20:386. doi: 10.1186/s12872-020-01654-3 (PMC7446172; doi:10.1186/s12872-020-01654-3)
Supplement: Supplementary file 1 — Additional file 1. [file 12872_2020_1654_MOESM1_ESM.docx]

Impact of plasma potassium normalization on short-term mortality in patients with hypertension and hypokalemia or low normal potassium

Running/short title Impact of correcting hypokalemia and borderline hypokalemia

Supplemental Tables 1-6

Supplemental Figures 1-3

Maria Lukács Krogager, MD^1^, Peter Søgaard, MD DMSc^1^, Christian Torp-Pedersen, MD DMSc^2^, Henrik Bøggild, MD^3,4^, Christina Ji-Young Lee, MD, PhD^2,8^, Anders Bonde, MD, PhD^8^, Jesper Q. Thomassen, MSc^9^, Gunnar Gislason, MD^5,6,7^, Manan Pareek, MD, PhD, FESC^2,10,11^, Kristian Kragholm, MD, PhD^1,4,12^

Affiliations:

*^1^ Department of Cardiology, Aalborg University Hospital, Aalborg, Denmark.*

*^2^ Department of Cardiology and Clinical Research, Nordsjællands Hospital, Hillerød, Denmark.*

*^3^ Public Health and Epidemiology Group, Department of Health Science and Technology, Aalborg University, Aalborg, Denmark.*

*^4^ Unit of Epidemiology and Biostatistics, Aalborg University Hospital, Aalborg, Denmark.*

*^5^ Department of Cardiology; Herlev and Gentofte University Hospital, Denmark.*

*^6^ The Danish Heart Foundation, Copenhagen, Denmark.*

*^7^ The National Institute of Public Health, University of Southern Denmark, Copenhagen, Denmark.*

*^8^ Department of Cardiology, Copenhagen University Hospital, Herlev and Gentofte, Hellerup, Denmark.*

*^9^Department of Clinical Biochemistry, Rigshospitalet, University of Copenhagen, Denmark.*

*^10^Department of Internal Medicine, Yale New Haven Hospital, Yale University School of Medicine, New Haven, United States of America*

*^11^Brigham and Women's Hospital, Heart & Vascular Center, Harvard Medical School, Boston, United States of America.*

*^12^Department of Cardiology, Regionshospital Nordjylland, Hjørring, Denmark.*

Corresponding author: Maria Lukács Krogager, Department of Cardiology, Hobrovej 18-22, 9000, Aalborg, Denmark. Tel: +45 53 14 88 24, Fax: + 45 97 66 44 80, E-mail: [lkcsmaria@yahoo.com](mailto:lkcsmaria@yahoo.com) [maria.krogager@rn.dk](mailto:maria.krogager@rn.dk).

Table S1. Nationwide registers used in the study

|  | **National Population Registry** | **National Patient Registry** | **National Prescription Registry** | **National Cause of Death Registry** | **The electronic registers of laboratory data** |
| --- | --- | --- | --- | --- | --- |
| **Created and established** | 1976 | 1977 | 1995 | 1970 | 1995 |
| **Examples of data in the registers** | Date of birth, gender, emigrations status | Hospital diagnoses, hospital contacts, procedure codes | Redeemed prescriptions for all Danish citizens | Date and cause of death | Serum/Plasma sodium, potassium and creatinine |
| **Coding** | N/A | ICD-10 | ATC-codes | ICD-10 | N/A |
| **Data used in the present study** | Age, gender and emigration status | Relevant comorbidities at baseline | Medication use at baseline | All-cause and presumed cardiovascular death (and follow-up time ) | Serum/Plasma sodium, potassium and creatinine |

Table S2. List of antihypertensive drugs and corresponding ATC codes used to define hypertension

| **ATC codes** | **Name of drug** |
| --- | --- |
| C02A | Antiadrenergic agents, centrally acting |
| C02B | Antiadrenergic agents, ganglion blocking |
| C02C | Antiadrenergic agents, peripherally acting |
| C02DA | Thiazide-derivatives |
| C02DB | Hydrazynophthalazin-derivatives |
| C02DD | Nitroferricyanide-derivatives |
| C02DG | Guanidin-derivatives |
| C02L | Antihypertensives and diuretics in combination |
| C03AA | Thiazides |
| C03AB | Thiazides and potassium in combination |
| C03BA | Sulfonamides |
| C03BB | Sulfonamides and potassium in combination |
| C03C | Loop diuretics |
| C03DA | Aldosteron antagonists |
| C03DB | Other potassium sparing agents |
| C03EA | Low-ceiling diuretics and potassium sparing agents |
| C03EB | High-ceiling diuretics and potassium sparing agents |
| C03X | Other diuretics |
| C07A | Beta-blockers |
| C07B | Beta-blockers and thiazides |
| C07C | Beta-blockers and other diuretics |
| C07D | Beta-blockers, thiazides and other diuretics |
| C07FB | Beta-blockers and calcium antagonists |
| C07FX | Beta-blockers and other combinations |
| C08C | Selective calcium antagonists primarily with vascular effect |
| C08D | Selective calcium antagonists with direct cardiac effect |
| C08E | Non-selective calcium antagonists |
| C08G | Calcium antagonists and diuretics |
| C09AA | Angiotensin converting enzyme inhibitors |
| C09BA | Angiotensin converting enzyme inhibitors and diuretics |
| C09BB | Angiotensin converting enzyme inhibitors and calcium antagonists |
| C09CA | Angiotensin II antagonists |
| C09DA | Angiotensin II antagonists and diuretics |
| C09DB | Angiotensin II antagonists and calcium antagonists |
| C09XA | Renin-inhibitors |

Table S3. Definitions of comorbidities, procedures and concomitant medications based on different ICD-10, Nordic Classification of Surgical Procedures (NCSP), and ATC codes identified prior to index date.

|  | **ICD-10 codes** | **Time prior to index date** | **NCSP codes** | **Time prior to index date** | **ATC codes** | **Time prior to index date** |
| --- | --- | --- | --- | --- | --- | --- |
| **Comorbidities and procedures** | | | | | | |
| Ischemic heart disease including myocardial infarction^1^ | I20-25 | 5 years | KFNG, KFNA-E | 5 years | − | − |
| Atrial flutter or fibrillation | I48 | 5 years | − | − | − | − |
| Second- or third- degree atrioventricular block | I44.1-.3 | 5 years | − | − | − | − |
| Ventricular tachycardia or fibrillation | I47.2, I49 | 5 years | − | − | − | − |
| Chronic obstructive pulmonary disease | J40-44 | 5 years | − | − | − | − |
| Chronic liver disease | B18, C22, K71-77 | 5 years | − | − | − | − |
| Syndrome of inappropriate antidiuretic hormone secretion | E22.2 | 5 years | − | − | − | − |
| Diabetes insipidus | E23.2, N25.1 | 5 years | − | − | − | − |
| Hypothyroidism | E00.1, E02-03, E89.0 | 5 years | − | − | − | − |
| Hyperthyroidism | DE050-59 | 5 years | − | − | − | − |
| Primary adrenal insufficiency | E27.1 | 5 years | − | − | − | − |
| Primary hyperaldosteronism | E26.0 | 5 years | − | − | − | − |
| Diabetes | E10-14 | 5 years | − | − | − | − |
| Cancer | C00-99 | 5 years | − | − | − | − |
| Hypertension | DI11-15 | 5 years | − | − | − | − |
| Heart failure | I110, I130, I132, I42, I50, J81 | 5 years | − | − | − | − |
| Stroke | DI61, DI62, DI63, DI64, DG458-459, DG433-438 | 5 years | − | − | − | − |
| Inflammatory bowel disease (IBD) | DK50-51 | 5 years | − | − | − | − |
| **Concomitant medications** | | | | | | |
| Potassium supplements | − | − | − | − | A12B  C03AB  C03BB  C03CB | 90 days |
| Loop diuretics | − | − | − | − | C03C | 90 days |
| Non-steroidal anti-inflammatory drugs | − | − | − | − | M01A | 90 days |
| Antimicrobials | − | − | − | − | J01CF06, J01CA01, J01CE, J01G, J02AA01,  J05AD01,  J01CF05 | 90 days |
| β2-agonists | − | − | − | − | R03AC02, C01CA2, C01CA24, R03AC13  C01CA02, R03AB02 R03CB01, R01BA02, R03AC03, R03AC12 | 90 days |
| Mineralo- and glucocorticoids | − | − | − | − | A01AC03, A07EA02, C05AA01, D07AA02, H02AB09, S01BA02, S02BA01, H02AA02, A07EA03, H02AB07 | 90 days |
| Laxatives | − | − | − | − | V03AE01, A06AB04, A06AG10 | 90 days |
| Xantines | − | − | − | − | R03DA04,  N06BC01 | 90 days |
| Macrolides | − | − | − | − | J01FA | 90 days |
| Trimethoprim | − | − | − | − | J01EA, J01EE | 90 days |

Table S4. Reference intervals for potassium in serum and plasma in different populations

| Population | US^1^ | German^2^ | Nordic^3^ |
| --- | --- | --- | --- |
| Serum reference intervals | 3.5-5.1 mmol/L | 3.7-5.1 mmol/L | 3.6-4.6 mmol/L |
| Plasma reference intervals | 3.4-4.8 mmol/L | 3.5-4.6 mmol/L | 3.5-4.4 mmol/L |

Table S5. Demographics stratified by survival status

|  |  | **Alive(n=8349)** | **Deceased (n=627)** | **Total (n=8976)** | **p-value** |
| --- | --- | --- | --- | --- | --- |
| Second potassium measurement | median(range) | 3.9(1.6, 7.1) | 3.9(1.5, 6.0) | 3.9(1.5, 7.1) | 0.23 |
| First potassium measurement | median(range) | 3.6(1.1, 3.7) | 3.5(1.8, 3.7) | 3.6(1.1, 3.7) | <0.01 |
| Age | median(range) | 67.7( 18.2, 100.8) | 75.1(27.9, 97.6) | 68.3( 18.2, 100.8) | <0.01 |
| Sex | Male | 3919 (46.9) | 301 (48.0) | 4,220 (47.0) | 0.63 |
| Renal insufficiency (second measuremt) |  | 497 (6.0) | 75 (12.0) | 572 (6.4) | <0.01 |
| Serum sodium (second measurement) | median(range) | 139(101, 161) | 137(105, 179) | 139(101, 179) | <0.01 |
| Renal insufficiency (first measuremt) |  | 520 (6.5) | 81 (13.4) | 601 (7.0) | <0.01 |
|  | missing | 336 | 22 | 358 |  |
| Hospitalization at the time of second potassium measurement |  | 6549 (78.5) | 602 (96.0) | 7,151 (79.7) | <0.01 |
| **Comorbidities** |  |  |  |  |  |
| Any malignancy |  | 1456 (17.4) | 249 (39.7) | 1,705 (19.0) | <0.01 |
| Chronic obstructive pulmonary disease |  | 1083 (13.0) | 152 (24.2) | 1,235 (13.8) | <0.01 |
| Chronic liver disease |  | 376 (4.5) | 58 (9.3) | 434 (4.8) | <0.01 |
| Inflammatory bowel disease |  | 147 (1.8) | 7 (1.1) | 154 (1.7) | 0.30 |
| Chronic kidney disease |  | 681 (8.2) | 55 (8.8) | 736 (8.2) | 0.64 |
| Diabetes |  | 1490 (17.8) | 96 (15.3) | 1,586 (17.7) | 0.12 |
| Hypertension (ICD-10) |  | 3138 (37.6) | 196 (31.3) | 3,334 (37.1) | <0.01 |
| Atrial fibrillation/Atrial flutter |  | 1524 (18.3) | 168 (26.8) | 1,692 (18.9) | <0.01 |
| Ischemic heart disease |  | 2087 (25.0) | 146 (23.3) | 2,233 (24.9) | 0.36 |
| Heart failure |  | 1667 (20.0) | 178 (28.4) | 1,845 (20.6) | <0.01 |
| Stroke |  | 987 (11.8) | 102 (16.3) | 1,089 (12.1) | <0.01 |
| **Pharmacotherapy** |  |  |  |  |  |
| Potassium supplement | ATC: A12B | 3989 (47.8) | 342 (54.5) | 4,331 (48.3) | <0.01 |
|  | ATC: C03 | 2248 (26.9) | 122 (19.5) | 2,370 (26.4) | <0.01 |
| Antimicrobials |  | 4674 (56.0) | 365 (58.2) | 5,039 (56.1) | 0.30 |
| Beta-2 agonists |  | 2011 (24.1) | 167 (26.6) | 2,178 (24.3) | 0.16 |
| Corticosteroids |  | 1767 (21.2) | 135 (21.5) | 1,902 (21.2) | 0.87 |
| Laxatives |  | 241 (2.9) | 17 (2.7) | 258 (2.9) | 0.90 |
| Xanthines |  | 237 (2.8) | 31 (4.9) | 268 (3.0) | <0.01 |
| NSAIDs |  | 4702 (56.3) | 353 (56.3) | 5,055 (56.3) | 1.00 |
| Calcium channel blockers |  | 2898 (34.7) | 174 (27.8) | 3,072 (34.2) | <0.01 |
| Beta blockers |  | 3571 (42.8) | 228 (36.4) | 3,799 (42.3) | <0.01 |
| Renin angiotensin system inhibitors |  | 5029 (60.2) | 262 (41.8) | 5,291 (58.9) | <0.01 |
| Mineral receptor antagonists |  | 1077 (12.9) | 176 (28.1) | 1,253 (14.0) | <0.01 |
| Thiazide diuretics |  | 3713 (44.5) | 164 (26.2) | 3,877 (43.2) | <0.01 |
| Loop diuretics |  | 3181 (38.1) | 406 (64.8) | 3,587 (40.0) | <0.01 |
| Potassium sparing diuretics |  | 206 (2.5) | 11 (1.8) | 217 (2.4) | 0.32 |
| Vasodilators |  | ≤3 | 0 (0.0) | ≤3 | 1.00 |
| Antiadrenergic drugs |  | 97 (1.2) | 5 (0.8) | 102 (1.1) | 0.52 |

Table S6. Sensitivity analyses. Potassium interval K: 3.8-4.0 mmol/L represented the reference range. Adjusted for age, gender, serum sodium, renal insufficiency, malignancy, heart failure, chronic liver disease, chronic obstructive pulmonary disease, diabetes mellitus, atrial flutter/fibrillation, stroke and ischemic heart disease, antihypertensive therapy, corticosteroids, antimicrobials, non-steroidal anti-inflammatory drugs, potassium supplement, xanthines, laxatives, digoxin.

|  |  | **Unadjusted** |  | **Adjusted** |  |  |
| --- | --- | --- | --- | --- | --- | --- |
| **1. Patients with normal kidney function (N= 8404 )** | **HR** | **95% CI** | **p-value** | **HR** | **95% CI** | **p-value** |
| P(K) 1.5-2.9 mmol/L | 2.83 | [1.91;4.20] | <0.001 | 2.33 | [1.56;3.46] | < 0.001 |
| P(K) 3.0-3.4 mmol/L | 1.40 | [1.06;1.86] | 0.02 | 1.35 | [1.02;1.79] | 0.04 |
| P(K) 3.5-3.7 mmol/L | 1.21 | [0.93;1.58] | 0.16 | 1.18 | [0.90;1.54] | 0.23 |
| P(K) 3.8-4.0 mmol/L | REF. |  |  |  |  |  |
| P(K) 4.1-4.6 mmol/L | 1.37 | [1.07;1.75] | 0.01 | 1.21 | [0.95;1.55] | 0.12 |
| P(K) 4.7-5.0 mmol/L | 2.68 | [1.85;3.87] | <0.001 | 2.29 | [1.58;3.32] | < 0.001 |
| P(K) 5.1-7.1 mmol/L | 5.68 | [3.62;8.90] | <0.001 | 2.81 | [1.76;4.47] | < 0.001 |
| **2. Patients without past history with malignancy (N= 7271)** |  |  |  |  |  |  |
| P(K) 1.5-2.9 mmol/L | 3.10 | [1.97;4.87] | <0.001 | 2.39 | [1.51;3.77] | < 0.001 |
| P(K) 3.0-3.4 mmol/L | 1.27 | [0.90;1.80] | 0.17 | 1.21 | [0.86;1.72] | 0.28 |
| P(K) 3.5-3.7 mmol/L | 1.17 | [0.85;1.62] | 0.32 | 1.16 | [0.84;1.60] | 0.36 |
| P(K) 3.8-4.0 mmol/L | REF. |  |  |  |  |  |
| P(K) 4.1-4.6 mmol/L | 1.23 | [0.91;1.66] | 0.17 | 1.05 | [0.78;1.42] | 0.74 |
| P(K) 4.7-5.0 mmol/L | 2.95 | [1.94;4.47] | <0.001 | 2.34 | [1.53;3.57] | < 0.001 |
| P(K) 5.1-7.1 mmol/L | 5.69 | [3.51;9.22] | <0.001 | 3.59 | [2.17;5.93] | < 0.001 |
| **3. Patients without past history with heart failure or loop diuretic prescription (N= 4882)** |  |  |  |  |  |  |
| P(K) 1.5-2.9 mmol/L | 3.79 | [2.06;7.00] | < 0.001 | 3.26 | [1.73;6.14] | <0.001 |
| P(K) 3.0-3.4 mmol/L | 1.68 | [1.05;2.66] | 0.03 | 1.64 | [1.02;2.63] | 0.04 |
| P(K) 3.5-3.7 mmol/L | 1.17 | [0.74;1.84] | 0.50 | 1.07 | [0.67;1.70] | 0.77 |
| P(K) 3.8-4.0 mmol/L | REF. |  |  |  |  |  |
| P(K) 4.1-4.6 mmol/L | 1.63 | [1.07;2.47] | 0.02 | 1.37 | [0.90;2.09] | 0.14 |
| P(K) 4.7-5.0 mmol/L | 2.98 | [1.52;5.83] | 0.001 | 2.12 | [1.07;4.18] | 0.03 |
| P(K) 5.1-7.1 mmol/L | 5.59 | [2.00;15.67] | 0.001 | 1.73 | [0.58;5.13] | 0.32 |
| **4. Patients without past history with ischemic heart disease (N= 6743)** |  |  |  |  |  |  |
| P(K) 1.5-2.9 mmol/L | 2.70 | [1.78;4.10] | <0.001 | 2.16 | [1.41;3.29] | < 0.001 |
| P(K) 3.0-3.4 mmol/L | 1.32 | [0.97;1.79] | 0.07 | 1.33 | [0.98;1.80] | 0.07 |
| P(K) 3.5-3.7 mmol/L | 1.27 | [0.96;1.68] | 0.09 | 1.30 | [0.98;1.72] | 0.07 |
| P(K) 3.8-4.0 mmol/L | REF. |  |  |  |  |  |
| P(K) 4.1-4.6 mmol/L | 1.37 | [1.05;1.79] | 0.02 | 1.23 | [0.94;1.61] | 0.13 |
| P(K) 4.7-5.0 mmol/L | 3.17 | [2.15;4.67] | <0.001 | 2.43 | [1.64;3.60] | < 0.001 |
| P(K) 5.1-7.1 mmol/L | 5.80 | [3.62;9.31] | <0.001 | 2.66 | [1.62;4.38] | < 0.001 |
| **5. Patients with P(K) 3.5-3.7 mmol/L at the first potassium measurement (N= 6111)** |  |  |  |  |  |  |
| P(K) 1.5-2.9 mmol/L | 3.82 | [2.24;6.53] | <0.001 | 2.16 | [1.25;3.73] | 0.006 |
| P(K) 3.0-3.4 mmol/L | 1.95 | [1.40;2.72] | <0.001 | 1.70 | [1.22;2.37] | 0.001 |
| P(K) 3.5-3.7 mmol/L | 1.16 | [0.84;1.59] | 0.36 | 1.09 | [0.79;1.49] | 0.61 |
| P(K) 3.8-4.0 mmol/L | REF. |  |  |  |  |  |
| P(K) 4.1-4.6 mmol/L | 1.26 | [0.95;1.68] | 0.11 | 1.06 | [0.80;1.42] | 0.68 |
| P(K) 4.7-5.0 mmol/L | 2.35 | [1.52;3.64] | <0.001 | 1.84 | [1.18;2.86] | 0.007 |
| P(K) 5.1-7.1 mmol/L | 5.54 | [3.40;9.03] | <0.001 | 2.81 | [1.68;4.71] | < 0.001 |
| **6. Patients with P(K)<3.5 mmol/L at the first potassium measurement (N= 2865)** |  |  |  |  |  |  |
| P(K) 1.5-2.9 mmol/L | 2.07 | [1.23;3.48] | 0.006 | 2.44 | [1.43;4.14] | 0.001 |
| P(K) 3.0-3.4 mmol/L | 0.73 | [0.46;1.18] | 0.20 | 0.89 | [0.55;1.44] | 0.65 |
| P(K) 3.5-3.7 mmol/L | 1.18 | [0.78;1.80] | 0.43 | 1.36 | [0.89;2.09] | 0.15 |
| P(K) 3.8-4.0 mmol/L | REF. |  |  |  |  |  |
| P(K) 4.1-4.6 mmol/L | 1.44 | [0.96;2.17] | 0.08 | 1.55 | [1.03;2.33] | 0.04 |
| P(K) 4.7-5.0 mmol/L | 3.70 | [2.16;6.32] | < 0.001 | 3.71 | [2.15;6.39] | < 0.001 |
| P(K) 5.1-7.1 mmol/L | 3.08 | [1.44;6.59] | 0.004 | 1.88 | [0.86;4.11] | 0.12 |
| **7. Last potassium measurement available within 6-100 days from the first potassium measurement (N= 8976)*** |  |  |  |  |  |  |
| P(K) 1.5-2.9 mmol/L | 1.74 | [1.31;2.29] | <0.001 | 1.41 | [1.06;1.86] | 0.02 |
| P(K) 3.0-3.4 mmol/L | 0.79 | [0.62;1.01] | 0.06 | 0.74 | [0.58;0.95] | 0.02 |
| P(K) 3.5-3.7 mmol/L | 0.74 | [0.58;0.95] | 0.02 | 0.68 | [0.53;0.87] | 0.002 |
| P(K) 3.8-4.0 mmol/L | REF. |  |  |  |  |  |
| P(K) 4.1-4.6 mmol/L | 0.88 | [0.64;1.21] | 0.44 | 0.88 | [0.64;1.21] | 0.43 |
| P(K) 4.7-5.0 mmol/L | 1.21 | [0.64;2.69] | 0.47 | 1.26 | [0.63;2.50] | 0.51 |
| P(K) 5.1-7.1 mmol/L | 2.36 | [0.75;7.46] | 0.14 | 2.11 | [0.77;5.77] | 0.15 |
| **8. Analyses perfomed on patients with time from first to second potassium measurement <=45 days (N= 8976)** |  |  |  |  |  |  |
| P(K) 1.5-2.9 mmol/L | 2.52 | [1.75;3.64] | <0.001 | 2.15 | [1.49;3.12] | < 0.001 |
| P(K) 3.0-3.4 mmol/L | 1.20 | [0.90;1.60] | 0.20 | 1.22 | [0.92;1.63] | 0.17 |
| P(K) 3.5-3.7 mmol/L | 1.20 | [0.93;1.56] | 0.15 | 1.20 | [0.93;1.55] | 0.16 |
| P(K) 3.8-4.0 mmol/L | REF. |  |  |  |  |  |
| P(K) 4.1-4.6 mmol/L | 1.16 | [0.91;1.47] | 0.24 | 1.10 | [0.87;1.41] | 0.43 |
| P(K) 4.7-5.0 mmol/L | 2.43 | [1.73;3.42] | <0.001 | 2.22 | [1.57;3.14] | < 0.001 |
| P(K) 5.1-7.1 mmol/L | 3.76 | [2.47;5.71] | <0.001 | 2.51 | [1.64;3.87] | < 0.001 |
| **9. Analyses perfomed on patients with time from first to second potassium measurement >45 days (N= 8976)** |  |  |  |  |  |  |
| P(K) 1.5-2.9 mmol/L | 2.21 | [0.28;17.67] | 0.45 | 2.20 | [0.26;18.87] | 0.47 |
| P(K) 3.0-3.4 mmol/L | 3.30 | [1.37;7.97] | 0.01 | 3.03 | [1.21;7.62] | 0.02 |
| P(K) 3.5-3.7 mmol/L | 1.13 | [0.41;3.12] | 0.81 | 1.23 | [0.44;3.44] | 0.69 |
| P(K) 3.8-4.0 mmol/L | REF. |  |  |  |  |  |
| P(K) 4.1-4.6 mmol/L | 2.88 | [1.27;6.53] | 0.01 | 2.22 | [0.96;5.10] | 0.06 |
| P(K) 4.7-5.0 mmol/L | 2.97 | [0.63;13.99] | 0.17 | 2.00 | [0.40;9.86] | 0.39 |
| P(K) 5.1-7.1 mmol/L | 11.43 | [2.43;53.82] | 0.002 | 2.46 | [0.39;15.61] | 0.34 |
| **10. Analyses performed on 3 predefined potassium intervals with 3.5-4.6 mmol/L as reference (8976)** |  |  |  |  |  |  |
| P(K) 1.5-3.4 mmol/L | 1.38 | [1.13;1.67] | 0.001 | 1.36 | [1.12;1.66] | 0.002 |
| P(K) 4.7-7.1 mmol/L | 2.80 | [2.19;3.57] | < 0.001 | 2.13 | [1.66;2.74] | < 0.001 |
| **11. Analyses performed on patients with available magnesium measurements at the time of plasma potassium draw (N=839)**** |  |  |  |  |  |  |
| P(K) 1.5-2.9 mmol/L | 2.53 | [1.11;5.74] | 0.03 | 2.46 | [1.05;5.74] | 0.04 |
| P(K) 3.0-3.4 mmol/L | 1.49 | [0.79;2.78] | 0.22 | 1.81 | [0.95;3.46] | 0.07 |
| P(K) 3.5-3.7 mmol/L | 1.20 | [0.66;2.19] | 0.54 | 1.05 | [0.57;1.94] | 0.87 |
| P(K) 3.8-4.0 mmol/L | REF. |  |  |  |  |  |
| P(K) 4.1-4.6 mmol/L | 1.54 | [0.88;2.71] | 0.13 | 1.39 | [0.78;2.48] | 0.26 |
| P(K) 4.7-5.0 mmol/L | 2.92 | [1.40;6.10] | 0.004 | 2.52 | [1.18;5.39] | 0.02 |
| P(K) 5.1-7.1 mmol/L | 2.69 | [1.08;6.69] | 0.04 | 1.98 | [0.75;5.22] | 0.17 |

*At the last available potassium measurement within 6-100 days the number of patients in each of the seven predefine potassium intervals was:

| P(K) 1.5-2.9 mmol/L | 781 |
| --- | --- |
| P(K) 3.0-3.4 mmol/L | 2986 |
| P(K) 3.5-3.7 mmol/L | 2855 |
| P(K) 3.8-4.0 mmol/L | 1314 |
| P(K) 4.1-4.6 mmol/L | 936 |
| P(K) 4.7-5.0 mmol/L | 84 |
| P(K) 5.1-7.1 mmol/L | 20 |

**At the time of first potassium measurement, 116 patients had hypomagnesemia (<0.7 mmol/L). Among patients with potassium concentrations below 3.5 mmol/L, 47 had hypomagnesemia at the time of first potassium measurement.

At the time of second potassium measurement, 109 patients had hypomagesemia, of which 42 had hypokalemia or borderline hypokalemia.

Figure S1. Population flowchart


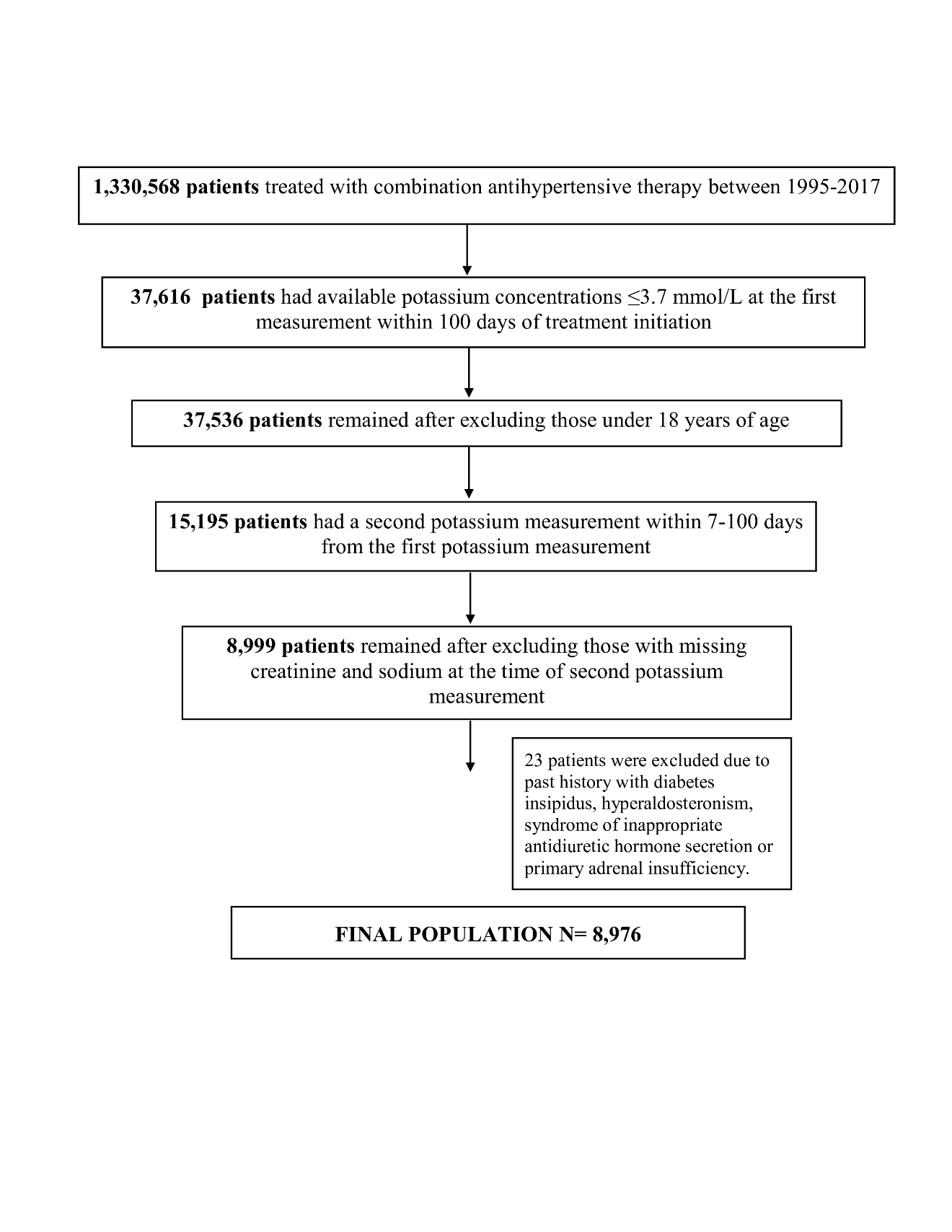


Figure S2. Distribution of the first potassium measurement (1, n=8976), average potassium measurements drawn within 1-5 days from the first potassium measurement (2, n=3490) and distribution of the second potassium measurement (3, n=8976).


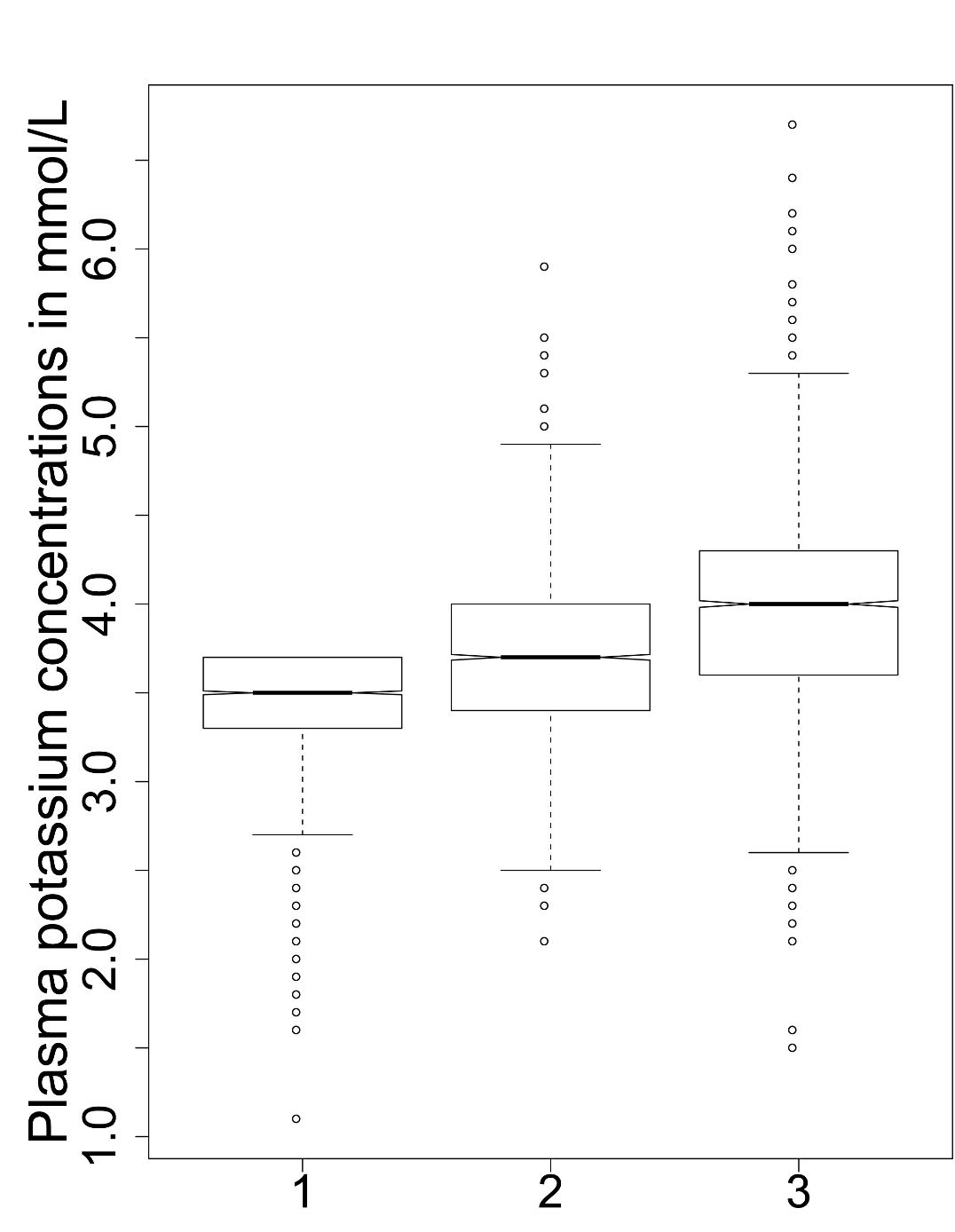


Figure S3. Kaplan Meier survival curves across the seven plasma potassium intervals. The p-values indicate the difference among plasma potassium groups compared to the reference group based on an unadjusted Cox regression model.


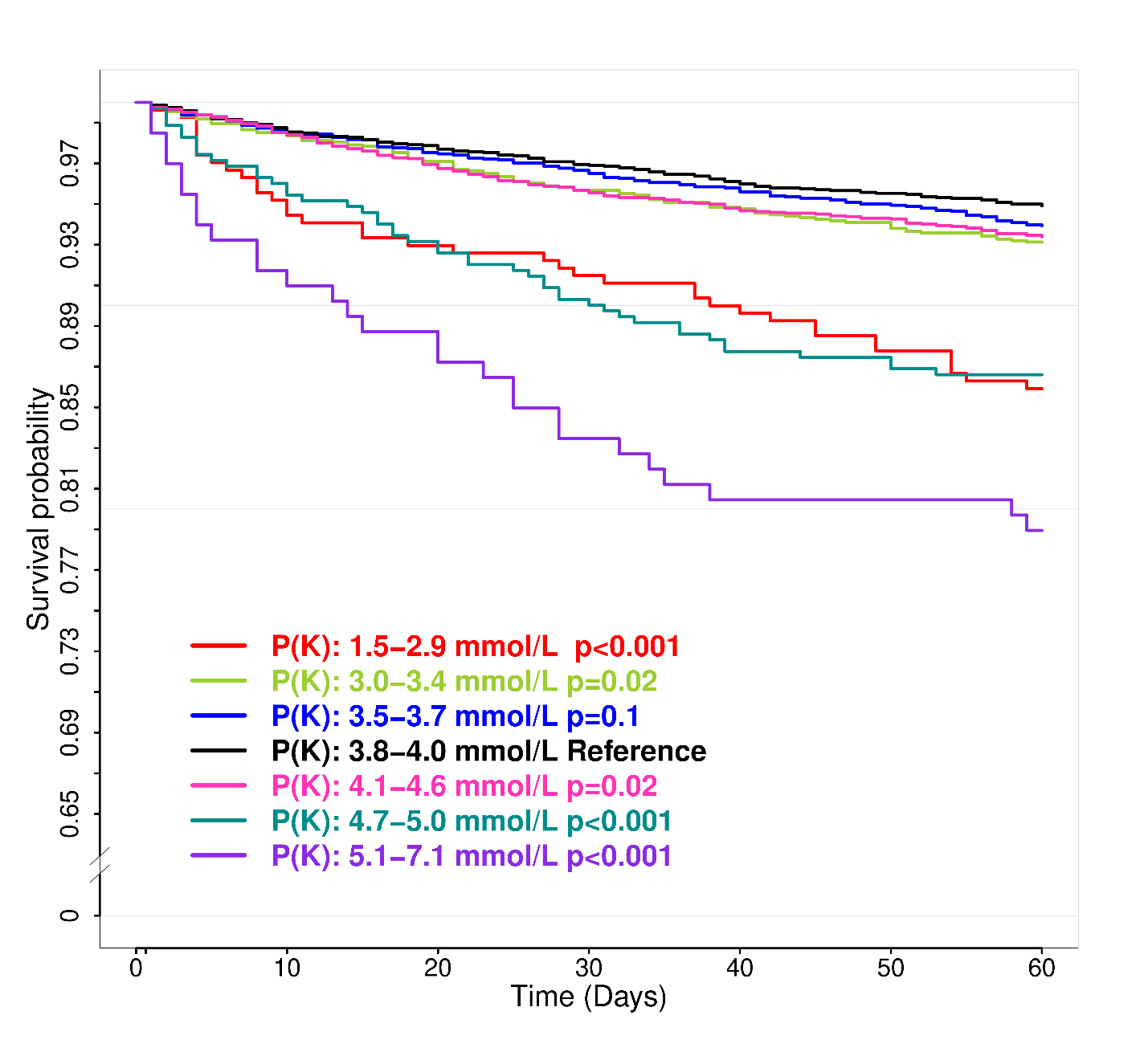


References

1. Burtis CA, Ashwood ER, Bruns DE. Tietz Textbook of Clinical Chemistry and Molecular Diagnostics. 5th ed. St. Louis, Missouri, USA: Elsevier Saunders; 2012.

2. Drogies T, Ittermann T, Lüdemann J, Klinke D, Kohlmann T, Lubenow N, Greinacher A, Völzke H, Nauck M. Potassium - Reference intervals for lithium-heparin plasma and serum from a population-based cohort. LaboratoriumsMedizin. 2010;34(1):39–44.

3. Rustad P, Felding P, Franzson L, Kairisto V, Lahti A, Martensson A, Hyltoft Petersen P, Simonsson P, Steensland H, Uldall A. The Nordic Reference Interval Project 2000: recommended reference intervals for 25 common biochemical properties. Scand J Clin Lab Invest. 2004;64(4):271–84.
